# Pulmonary Embolism in COVID-19 Patients: Which Diagnostic Algorithm Should We Use?

**DOI:** 10.3389/fcvm.2021.714003

**Published:** 2021-08-13

**Authors:** Angelo Porfidia, Carolina Mosoni, Rosa Talerico, Enrica Porceddu, Andrea Lupascu, Paolo Tondi, Francesco Landi, Roberto Pola

**Affiliations:** ^1^Department of Medicine, Fondazione Policlinico Universitario A. Gemelli IRCCS, Università Cattolica del Sacro Cuore, Rome, Italy; ^2^Division of Angiology, Fondazione Policlinico Universitario A. Gemelli IRCCS, Università Cattolica del Sacro Cuore, Rome, Italy; ^3^Section of Internal Medicine and Thromboembolic Diseases, Fondazione Policlinico Universitario A. Gemelli IRCCS, Università Cattolica del Sacro Cuore, Rome, Italy

**Keywords:** COVID-19, pulmonary embolism, computed tomography pulmonary angiography, diagnosis, algorithm

## Abstract

**Introduction:** Although pulmonary embolism (PE) is a frequent complication of the clinical course of COVID-19, there is a lack of explicit indications regarding the best algorithm for diagnosing PE in these patients. In particular, it is not clear how to identify subjects who should undergo computed tomography pulmonary angiography (CTPA), rather than simply X-ray and/or high resolution computed tomography (HRCT) of the chest.

**Methods:** We retrospectively analyzed COVID-19 patients who presented to the Emergency Department (ED) of our University hospital with acute respiratory failure, or that developed acute respiratory failure during hospital stay, to determine how many of them had a theoretical indication to undergo CTPA for suspected PE according to current guidelines. Next, we looked for differences between patients who underwent CTPA and those who only underwent X-ray and/or HRCT of the chest. Finally, we determined whether patients with a confirmed diagnosis of PE had specific characteristics that made them different from those with a CTPA negative for PE.

**Results:** Out of 93 subjects with COVID-19 and acute respiratory failure, 73 (78.4%) had an indication to undergo CTPA according to the revised Geneva and Wells scores and the PERC rule-out criteria, and 54 (58%) according to the YEARS algorithm. However, in contrast with these indications, only 28 patients (30.1%) underwent CTPA. Of note, they were not clinically different from those who underwent X-ray and/or HRCT of the chest. Among the 28 subjects who underwent CTPA, there were 10 cases of PE (35.7%). They were not clinically different from those with CTPA negative for PE.

**Conclusions:** COVID-19 patients with acute respiratory failure undergo CTPA, X-ray of the chest, or HRCT without an established criterion. Nonetheless, when CTPA is performed, the diagnosis of PE is anything but rare. Validated tools for identifying COVID-19 patients who require CTPA for suspected PE are urgently needed.

## Introduction

Coronavirus disease 2019 (COVID-19) represents an unprecedented threat to global health care systems. The severity of COVID-19 infection varies between a mild respiratory disease to acute respiratory distress syndrome, shock, and multi-organ failure associated with an increased risk of death. The clinical course of COVID-19 is often accompanied by a hyperinflammatory response and systemic coagulation derangement, which may evolve into overt disseminated intravascular coagulopathy (DIC). The systemic activation of blood coagulation and pulmonary thrombo-inflammation with local vascular damage caused by COVID-19 and other important risk factors, such as reduced mobility, may increase the risk of deep vein thrombosis (DVT) and pulmonary embolism (PE) (1–4). Nonetheless, there is a lack of explicit indications regarding the best algorithm for diagnosing PE in COVID-19 patients (5). In particular, it is not clear whether the most recent guidelines for the diagnosis and management of acute PE, that have been developed in 2019 by the European Respiratory Society and the European Society of Cardiology (6), may be applied with success to COVID-19 patients with respiratory insufficiency. These guidelines consider the use of the revised Geneva and Wells pre-test probability scores (7, 8) a key step in the diagnosis of PE. They also suggest the use of algorithms meant to identify patients with low likelihood of having PE, such as the PE Rule-out Criteria (PERC) (9) and the YEARS clinical decision rule (10). Also for these algorithms it is not clear whether they perform well in the COVID-19 population (11).

In this study, we conducted a retrospective analysis of a cohort of patients hospitalized for COVID-19 and acute respiratory failure, to determine how many of them had an indication to undergo computed tomography pulmonary angiography (CTPA) according to guideline-recommended algorithms for diagnosing PE. We also looked for differences between patients who underwent and did not undergo CTPA, to understand whether diagnostic workup for PE in COVID-19 patients is based on established criteria. Finally, we determined whether patients with a confirmed diagnosis of PE had specific characteristics that made them different from those with a CTPA negative for PE.

## Methods

We retrospectively analyzed patients admitted for COVID-19 to our University Hospital during two random periods of the year 2020 (March 15th—April 10th and October 11th—November 27th), as part of protocol number 0018324/21, approved by the Ethics Committee of the Fondazione Policlinico Universitario A. Gemelli IRCCS on 19 May 2021. Inclusion criteria were: infection by SARS-CoV-2 confirmed by positive molecular assay on oral/nasopharyngeal swabs; hospitalization in non-intensive care unit (ICU) medical wards; presence of acute respiratory failure at the time of hospital admission or during hospital stay. Acute respiratory failure was defined as sudden need for oxygen supplementation in patients who did not require oxygen therapy before. All patients were on treatment with a prophylactic dose of anticoagulant since their arrival in the hospital, as established by our internal guidelines for the prevention of VTE in COVID-19 patients. Exclusion criteria were: age <18 years; full-dose anticoagulant therapy for conditions such as atrial fibrillation and/or previous venous thromboembolism (VTE). Data are presented as mean ± standard deviation (SD) or number and percentage (%). Differences between groups were analyzed by Chi square test or Fisher's exact test for dichotomous variables and by Mann-Whitney test for continuous variables. Differences were considered statistically significant for *P* < 0.05.

## Results

**Figure 1:**
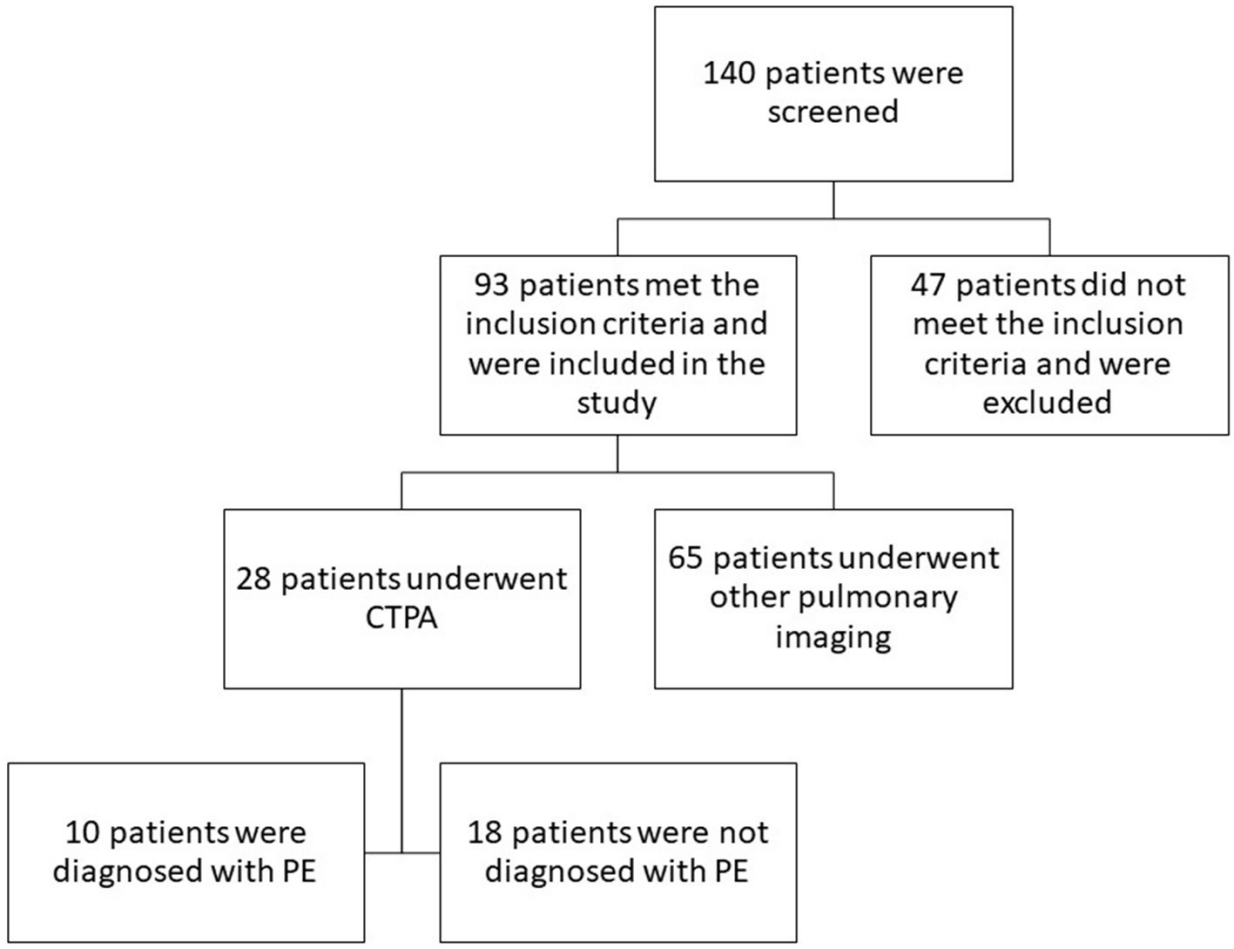
Study flowchart.

One hundred forty patients were screened. Among them, 93 patients met the criteria to be included in the analysis (Figure 1). Their demographical and clinical characteristics are presented in Table 1. They were mostly males (77.4%) with a mean age of 68.0 ± 14.2 years. Many of them had hypertension (61.3%), diabetes (20.4%), ischemic cardiovascular diseases (20.4%), and obesity (20.4%). Many had relevant risk factors for PE, including reduced mobility (80.6%), cancer (12.9%), previous VTE (6.4%), and a recent (within 1 month) trauma and/or surgery (4.3%). Most patients had D-dimer levels above the age-adjusted range of normality, at least once during hospital stay (78.4%). Extremely high levels of D-dimer (>5,000 μg/L) were found in a relevant portion of patients (14.0%) during hospitalization.

**Table 1:** Characteristics of the patients and theoretical indication to undergo CTPA according to Geneva score, Wells score, PERC, and YEARS algorithm.

	**Total (*n* = 93)**	**CTPA^a^ (*n* = 28)**	**Other^b^ pulmonary imaging (*n* = 65)**	***P***
Age—years ± SD	68.8 ± 14.2	68.1 ± 11.8	68.9 ± 15.2	0.83
Male gender—*n* (%)	72 (77.4)	24 (85.7)	48 (73.8)	0.28
Hypertension—*n* (%)	57 (61.3)	15 (53.6)	42 (64.6)	0.32
Diabetes mellitus—*n* (%)	19 (20.4)	5 (17.9)	14 (21,5)	0.78
Ischemic CV diseases (CAD, stroke, TIA, PAD)—*n* (%)	19 (20.4)	2 (7.1)	17 (26.2)	0.05
Obesity—*n* (%)	19 (20.4)	4 (14.3)	15 (23.1)	0.41
Previous history of VTE—*n* (%)	6 (6.4)	2 (7.1)	4 (6.2)	1
Previous or active malignancy—*n* (%)	12 (12.9)	4 (14.3)	8 (12.3)	0.75
Reduced mobility—*n* (%)	75 (80.6)	23 (82.1)	52 (80.0)	0.81
Recent trauma/surgery—*n* (%)	4 (4.3)	3 (10.7)	1 (1.5)	0.08
D-dimer > age-adjusted range of normality in the ED—*n* (%)	64 (68.8)	23 (82.1)	41 (63.1)	0.07
D-dimer > age-adjusted range of normality at least once during hospital stay—*n* (%)	73 (78.5)	24 (85.7)	49 (75.4)	0.41
D-dimer > 1,000 ng/ml at least once during hospital stay—*n* (%)	55 (59.1)	20 (71.4)	35 (53.8)	0.11
D-dimer > 5,000 ng/ml at least once during hospital stay—*n* (%)	13 (14)	6 (21.4)	7 (10.8)	0.17
D-dimer > 10,000 ng/ml at least once during hospital stay—*n* (%)	11 (11.8)	5 (17.9)	6 (9.2)	0.3
Indication to CTPA according to Geneva score for PE in combination with D-dimer—*n* (%)	73 (78.5)	24 (85.7)	49 (75.4)	0.41
Indication to CTPA according to Wells score for PE in combination with D-dimer—*n* (%)	73 (78.5)	24 (85.7)	49 (75.4)	0.41
Indication to CTPA according to PERC—*n* (%)	73 (78.5)	24 (85.7)	49 (75.4)	0.41
Indication to CTPA according to YEARS algorithm—*n* (%)	54 (58.1)	20 (71.4)	34 (52.3)	0.08

a *Patients who underwent CTPA*.

b *Patients who underwent other pulmonary imaging*.

We tested our patients with the revised Geneva and Wells score, in combination with D-dimer levels, as recommended by international guidelines (6), to determine how many of them had a theoretical indication to undergo CTPA for suspected PE. We found that there were 73 subjects (78.4%) with this indication. We evaluated whether it was possible to reduce this number utilizing the PERC. Nonetheless, we found that the same 73 patients still had a theoretical indication to undergo CTPA. Then, we used the YEARS clinical decision rule to select patients without need for diagnostic workup for PE. However, 54 patients (58.1%) still had indication to undergo CTPA. These results are presented in Table 1.

In contrast with these results, we found that in our population only 28 patients (30.1%) underwent CTPA, while the remaining patients underwent either X-ray and/or HRCT of the chest. To assess whether these patients underwent CTPA for a specific reason, we compared their clinical characteristics to those of the patients that did not undergo CTPA (Table 1). We found no differences in terms of age, gender, comorbidities, D-dimer levels, pre-test probability scores, and rule-out criteria.

Finally, we determined that, among the 28 patients who underwent CTPA in our population, 10 had PE, with a diagnostic yield of 35.7%. We evaluated whether patients with positive CTPA were different from those with negative CTPA (Table 2). However, we did not find statistically significant differences between these two groups, in terms of age, gender, clinical presentation, and comorbidities with the exception of D-dimer >1,000 ng/ml and the indication to CTPA based on the YEARS algorithm (*p* = 0.03). Nonetheless, there were many patients without confirmed PE who had D-dimer >1,000 ng/ml and an indication to undergo CTPA based on the YEARS algorithm (61.1%).

**Table 2:** Demographical and clinical characteristics of the patients with CTPA positive for PE and of those with CTPA negative for PE.

	**CTPA positive for PE (*n* = 10)**	**CTPA negative for PE (*n* = 18)**	***P***
Age—years ± SD	73.0 ± 13.1	65.0 ± 10.2	0.06
Male gender—*n* (%)	9 (90.0)	15 (8.3)	1
Hypertension—*n* (%)	7 (70.0)	8 (44.4)	0.25
Diabetes mellitus—*n* (%)	2 (20.0)	3 (16.7)	1
Ischemic CV diseases (CAD, stroke, TIA, PAD)—*n* (%)	2 (20.0)	0 (0.0)	—
Obesity—*n* (%)	2 (20.0)	2 (11.1)	0.6
Previous history of VTE—*n* (%)	1 (10.0)	1 (5.6)	1
Previous or active malignancy—*n* (%)	1 (10.0)	3 (16.7)	1
Reduced mobility—*n* (%)	9 (90.0)	14 (77.8)	0.63
Recent trauma/surgery—*n* (%)	2 (20.0)	1 (5.6)	0.28
D-dimer > age-adjusted range of normality in the ED—*n* (%)	10 (100.0)	13 (72.2)	0.13
D-dimer > age-adjusted range of normality during hospital stay—*n* (%)	10 (100.0)	14 (77.8)	0.27
D-dimer>1,000 ng/ml at least once during hospital stay—*n* (%)	10 (100.0)	11 (61.1)	0.03
D-*dimer*>5,000 ng/ml at least once during hospital stay—*n* (%)	4 (40.0)	2 (11.1)	0.15
D-dimer>10,000 ng/ml at least once during hospital stay—*n* (%)	3 (30.0)	2 (11.1)	0.31
Indication to CTPA according to Geneva score for PE—no. (%)	10 (100.0)	14 (77.8)	0.27
Indication to CTPA according to Wells criteria for PE—no. (%)	10 (100.0)	14 (77.8)	0.27
Indication to CTPA according to PERC—no. (%)	10 (100.0)	14 (77.8)	0.27
Indication to CTPA according to YEARS algorithm—*n* (%)	10 (100.0)	11 (61.1)	0.03

## Discussion

In this study we tested four distinct guideline-recommended algorithms for diagnosing or ruling out PE in a population of subjects with COVID-19 and acute respiratory failure. Regardless the algorithm, the proportion of patients with a theoretical indication to undergo CTPA was much higher than the number of patients who underwent the exam in real-life. This might indicate that current recommendations for diagnosing PE overestimate the need for CTPA in the COVID-19 population. Alternatively, it might indicate that physicians do not properly follow the guidelines for the diagnosis of PE when managing COVID-19 patients. In either case, the result is that patients undergo CTPA without an established criterion. Indeed, in our study the subjects who underwent CTPA were not different to those who did not. This point seems to indicate that, regardless the well-established relationship between COVID-19 patients and VTE (12), clinicians tend to do not request CTPA, even in presence of high pre-test probability of PE. This is probably due to the fact that, in these patients, there is an alternative reason that might explain the acute respiratory failure, i.e., COVID-19 pneumonia. Nonetheless, when these patients undergo CTPA, a diagnosis of PE is anything but rare. Indeed, in our cohort, 10 out of 28 CTPA were positive for PE. Consistent with this concept is the recent evidence that, when consecutive patients hospitalized with a diagnosis of COVID-19 undergo CTPA at the time of hospital admission, PE is diagnosed in about 1 patient out of 7 CTPA (13).

Our study has some limitations, including a retrospective single-center design, a small sample size and low number of patients who underwent CTPA. Also, it should be noted that the algorithms that we used have been validated mainly for patients who are not receiving anticoagulants, while in our study all subjects were already receiving pharmacological thromboprophylaxis. Another potential limitation is the fact that we only analyzed patients hospitalized during two randomly selected period of 2020 and they might not be fully representative of all COVID-19 patients.

The absence of established algorithms for the diagnosis of PE in subjects with COVID-19 is a serious limitation to the correct clinical and therapeutic management of these patients. Validated tools for identifying COVID-19 patients who require CTPA are urgently needed.

## Data Availability Statement

The raw data supporting the conclusions of this article will be made available by the authors, without undue reservation.

## Ethics Statement

The studies involving human participants were reviewed and approved by Fondazione Policlinico Universitario A. Gemelli IRCCS. Written informed consent for participation was not required for this study in accordance with the national legislation and the institutional requirements.

## Author Contributions

AP and RP contributed to conception and design of the study. CM, RT, and EP organized the database. CM performed the statistical analysis. AP, CM, PT, FL, and RP wrote the first draft of the manuscript. All authors contributed to manuscript revision, read, and approved the submitted version.

## Conflict of Interest

The authors declare that the research was conducted in the absence of any commercial or financial relationships that could be construed as a potential conflict of interest.

## Publisher's Note

All claims expressed in this article are solely those of the authors and do not necessarily represent those of their affiliated organizations, or those of the publisher, the editors and the reviewers. Any product that may be evaluated in this article, or claim that may be made by its manufacturer, is not guaranteed or endorsed by the publisher.

## References

[1] Tang N Li D Wang X Sun Z. Abnormal coagulation parameters are associated with poor prognosis in patients with novel coronavirus pneumonia. J Thromb Haemost. (2020) 18:844–7. 10.1111/jth.14768 32073213 PMC7166509

[2] Al-Samkari H Karp Leaf RS Dzik WH Carlson JCT Fogerty AE Waheed A. COVID-19 and coagulation: bleeding and thrombotic manifestations of SARS-CoV-2 infection. Blood. (2020) 136:489–500. 10.1182/blood.2020006520 32492712 PMC7378457

[3] Porfidia A Porceddu E Talerico R Montalto M Landi F Pola R. Second wave of the COVID-19 pandemic: D-dimer levels are not so high anymore. J Thromb Thrombolysis. (2021). 10.1007/s11239-021-02454-y. [Epub ahead of print]. 33886038 PMC8060786

[4] Wang T Chen R Liu C Liang W Guan W Tang R. Attention should be paid to venous thromboembolism prophylaxis in the management of COVID-19. Lancet Haematol. (2020) 7:e362–3. 10.1016/S2352-3026(20)30109-5 32278361 PMC7158946

[5] Porfidia A Talerico R Mosoni C Porceddu E Pola R. CT pulmonary angiography for the diagnosis of pulmonary embolism in patients with COVID-19: when, why, and for who? Radiology. (2021) 299:E287. 10.1148/radiol.2021210400 33754832 PMC8026115

[6] Konstantinides SV Meyer G Becattini C Bueno H Geersing GJ Harjola VP. 2019. ESC Guidelines for the diagnosis and management of acute pulmonary embolism developed in collaboration with the European Respiratory Society (ERS). Eur Heart J. (2020) 41:543–603. 10.1093/eurheartj/ehz405 31504429

[7] Le Gal G Righini M Roy PM Sanchez O Aujesky D Bounameaux H. Prediction of pulmonary embolism in the emergency department: the revised Geneva score. Ann Intern Med. (2006) 144:165–71. 10.7326/0003-4819-144-3-200602070-00004 16461960

[8] Wells PS Ginsberg JS Anderson DR Kearon C Gent M Turpie AG. Use of a clinical model for safe management of patients with suspected pulmonary embolism. Ann Intern Med. (1998) 129:997–1005. 10.7326/0003-4819-129-12-199812150-00002 9867786

[9] van der Hulle T Cheung WY Kooij S Beenen LFM van Bemmel T van Es J. Simplified diagnostic management of suspected pulmonary embolism (the YEARS study): a prospective, multicentre, cohort study. Lancet. (2017) 390:289–97. 10.1016/S0140-6736(17)30885-1 28549662

[10] Kline JA Courtney DM Kabrhel C Moore CL Smithline HA Plewa MC. Prospective multicenter evaluation of the pulmonary embolism rule-out criteria. J Thromb Haemost. (2008) 6:772–80. 10.1111/j.1538-7836.2008.02944.x 18318689

[11] Porfidia A Pola E Pola R. Prevalence of pulmonary embolism on hospital admission in COVID-19 patients: is there a role for pre-test probability scores and home treatment? Eur Respir J. (2021). 10.1183/13993003.00785-2021. [Epub ahead of print]. 33833035 PMC8034056

[12] Porfidia A Valeriani E Pola R Porreca E Rutjes AWS Di Nisio M. Venous thromboembolism in patients with COVID-19: systematic review and meta-analysis. Thromb Res. (2020) 196:67–74. 10.1016/j.thromres.2020.08.020 32853978 PMC7420982

[13] Jevnikar M Sanchez O Chocron R Andronikof M Raphael M Meyrignac O. Prevalence of pulmonary embolism in patients with COVID 19 at the time of hospital admission. Eur Respir J. (2021) 58:2100116. 10.1183/13993003.00116-2021 33692122 PMC7947356

